# Safety and efficacy of intra-articular infiltration of purified autologous adipose tissue for osteoarthritis treatment: a pre-post study

**DOI:** 10.1186/s40634-022-00534-3

**Published:** 2022-09-27

**Authors:** Félix López

**Affiliations:** 1 Maestranza Medical Center, Madrid, Spain; 2 Bluehealthcare, Madrid, Spain

**Keywords:** Osteoarthritis, Knee, Cartilage, Regenerative medicine, Adipose tissue, Mesenchymal stem cells, Lipocell

## Introduction

Osteoarthritis (OA) is a degenerative cartilage disease that affects joints, causing pain and disability. Conservative treatments, such as physiotherapy or non-steroidal anti-inflammatory drugs, are possible during the early phase of OA, but their efficacy is limited. Besides, OA’s last stage is characterized by unbearable pain and massive cartilage disruption, thus requiring a surgery solution (i.e., joint replacement).

The gap between these two phases is huge, but there are no definitive solutions for a complete symptomatic resolution. Intra-articular administration of hyaluronic acid, as well as platelet-rich-plasma (PRP) treatments, which exploit the use of autologous growth factors [1], can offer relief in OA patients [2]. In addition, knee arthroscopy has no more benefits than sham surgery in OA [3], although it is the best technique to repair ligaments and menisci, which, if damaged, can accelerate OA degeneration. Therefore, the ideal conservative treatment should be efficient not only in treating OA symptoms, but in preventing its progression and worsening as well. An early-onset OA means an early joint replacement, which is an option that should be postponed as much as possible due to its invasiveness.

Regenerative medicine can be a conservative and optimal solution for OA treatment. Mesenchymal stem cells (MSCs) can release growth factors and anti-inflammatory cytokines that promote tissue regeneration [4]. MSCs can be found in many tissues, and adipose tissue has been recently discovered as an optimal MSC source because of its ease in harvesting and its abundance compared to the bone marrow [5]. In this regard, some in vitro and animal studies showed that MSCs can promote cartilage repair by exerting a paracrine effect [6]. Moreover, some clinical studies have shown that the isolation, culture, and expansion of MSCs from adipose tissue, along with their subsequent intra-articular injection can safely ameliorate pain symptoms and improve knee functionality in patients suffering knee OA [3, 7–9]. In addition, intra-articular injections of autologous adipose tissue seem to be beneficial for function improvement and pain relief in knee OA [10, 11].

Adipose-derived MSCs (AD-MSCs) can be obtained by using medical devices that comply with minimal manipulation requirements set by European and United States regulatory agencies, and many authors have demonstrated their safety and efficacy [12–15]. However, most of these studies did not include patients with the most advanced grade of knee OA (i.e., grade IV of the Kellgren-Lawrence classification [16]) or only included a small number of them. Therefore, the aim of this study was to analyze the clinical follow-up of patients with knee OA, including those with grade IV OA, treated with intra-articular injections of purified autologous micro-fragmented adipose tissue obtained with the medical device Lipocell.

## Materials and methods

### Study design and patients

This was an analysis of patients with symptomatic knee OA who underwent an intra-articular injection of purified adipose tissue between March 2018 and July 2019 at two centers in Madrid: Maestranza Medical Center and Beata María Ana Hospital. Individuals aged between 40 and 80 years old, diagnosed with unilateral or bilateral knee OA according to the American College of Rheumatology criteria [17] and the Kellgren-Lawrence classification [16] who had not received any surgery or infiltration within the previous three months were included in the study. Other inclusion criteria were a Visual Analogue Scale (VAS) score equal to or higher than 2.5, a body mass index (BMI) lower than 35 kg/m^2^, and willingness to be followed up for nine months. Patients with a severe knee varus/valgus deformity (i.e., higher than 15°), an autoimmune disease, or a rheumatic illness were excluded. All patients signed an informed consent form to participate in the study, which followed the ethical standards of the 1964 Helsinki Declaration and its later amendments.

### Procedures

All patients underwent minor liposuction to collect abdominal adipose tissue. The procedure consisted of infiltration of Klein solution (lidocaine 2% and adrenaline 1 mg/mL in 500 mL NaCl 0.9% solution) into the abdominal subcutaneous fat, followed by liposuction with fenestrated blunt cannulae and collection of approximately 60 mL of lipoaspirated fat. Then, the lipoaspirate was processed with Lipocell (Tiss’You, RSM), a Class II-a medical device, following the manufacturer’s instructions. Briefly, the lipoaspirate was introduced in the device, where it was dialyzed with a filter and washed with 300 mL-500 mL of NaCl 0.9%. In roughly 5 min, this device allows to eliminate undesired products while preserving AD-MSCs viability and doubling the AD-MSCs yield with low manipulation [18]. Subsequently, the lipoaspirate was recovered with a 10 mL syringe, 6 mL of purified adipose tissue were infiltrated in the knee with an 18 G needle, and flexion–extension movements were performed to diffuse the product into the joint. Patients with a meniscal tear were treated with knee arthroscopy for lavage and debridement of loose bodies before injecting the purified lipoaspirated fat. All patients were recommended not to take any non-steroidal anti-inflammatory drugs (NSAIDs) within the three weeks before the procedure.

Patients were discharged the same day of the infiltration. If necessary, the pain was controlled with ice and acetaminophen; no ibuprofen or any other NSAIDs were allowed. Elevation and flexion of the knee were recommended since the first day, and partial weight-bearing with the help of crutches was suggested for two weeks, always depending on the patient’s pain. Afterward, patients began to perform eccentric exercises supervised by a physiotherapist for one month. Low-impact sports (e.g., cycling, walking, swimming) and high-impact physical activity were allowed six-to-eight weeks and three months after the procedure, respectively.

### Variables and measurements

The analyzed variables included age, sex, BMI, and Kellgren-Lawrence classification of OA. In addition, the VAS score and Western Ontario and McMaster Universities Osteoarthritis Index (WOMAC) were used to assess pain and functionality, respectively, before the procedure and at three, six, and nine months after treatment.

### Statistical analyses

Categorical variables were presented as frequency and percentages, and quantitative variables as the mean and standard deviation (SD).

WOMAC and VAS score differences among the different times evaluated were assessed using the Wilcoxon signed-rank test. The threshold for statistical significance was set at a two-sided alpha value of 0.05. All analyses were performed with GraphPad Prism v.9.

## Results

A total of 24 patients were enrolled, 15 (62.5%) women and 9 (37.5%) men, with a mean (SD) of 63.8 (11.4) years and a mean (SD) BMI of 28.3 (5.1) kg/m^2^. According to the Kellgren-Lawrence classification, 3 (12.5%), 10 (41.7%), and 11 (45.8%) patients presented Grade 2, Grade 3, and Grade 4 OA, respectively. In addition, 14 (58.3%) patients underwent knee arthroscopy for lavage and debridement of loose bodies before injecting the purified lipoaspirated fat.

**Fig. 1:**
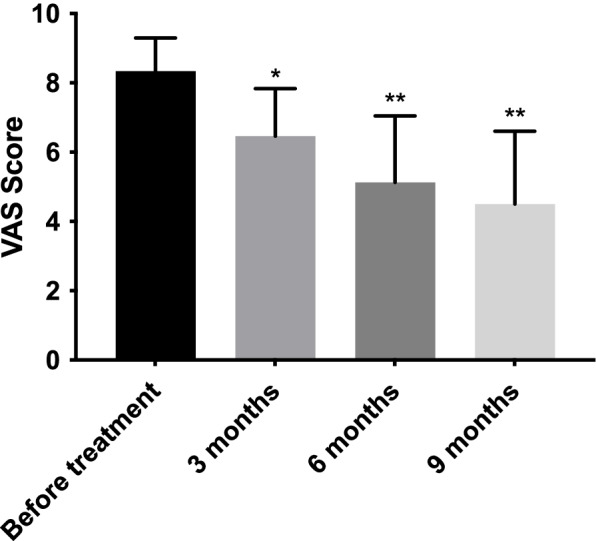
Mean functional Visual Analogue Scale (VAS) scores (*n* = 24) before and after Lipocell treatment. Error bars show standard deviation. *p vs. before treatment < 0.05; **p vs. before treatment < 0.01

**Fig. 2:**
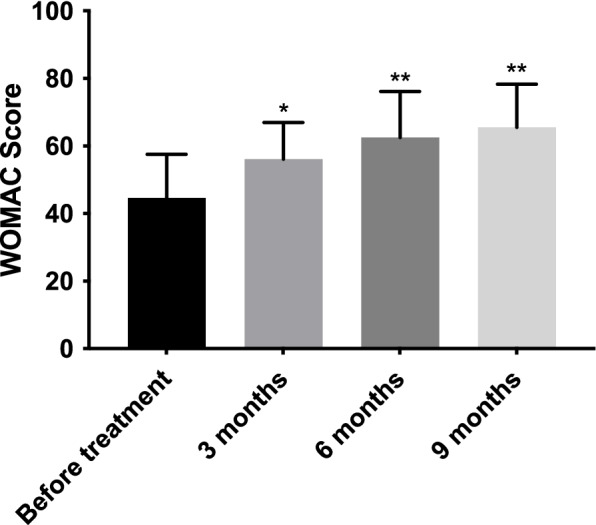
Mean functional Western Ontario and McMaster Universities Arthritis Index (WOMAC) scores (*n* = 24) before and after Lipocell treatment. Error bars show standard deviation. *p vs. before treatment < 0.05; **p vs. before treatment < 0.01

Patients’ pain decreased progressively over time, with a VAS mean (SD) score of 8.3 (1.0) before treatment, and 6.5 (1.4), 5.1 (1.9), and 4.5 (2.1) at three, six, and nine months after treatment, respectively (Fig. 1). Conversely, functionality increased over time; WOMAC mean (SD) scores were 44.6 (12.9) before the procedure, 56.1 (10.9) at three months, 62.5 (13.6) at six months, and 65.5 (12.8) at nine months (Fig. 2). Moreover, there were no adverse events reported in this study.

## Discussion

In this study, patients with knee OA who underwent an intra-articular infiltration of purified autologous adipose tissue in the knee—alone, or after an arthroscopy for the lavage and debridement of loose bodies in case of meniscal tear—experienced less pain and improved functionality after three, six, and nine months of follow-up.

Several studies indicate that intra-articular administration of purified autologous micro-fragmented adipose tissue is a safe and effective treatment for improving knee OA symptoms [12–15]. However, it is an emerging topic, and all reports about the safety and performance of this novel treatment are pivotal. Moreover, most of these studies did not include, or only included a small proportion of, patients with grade IV knee OA, whereas almost half of the patients in my study presented a grade IV knee OA, the most advanced stage according to Kellgren-Lawrence [16].

Unlike most studies, in this analysis, 14 out of 24 patients underwent knee arthroscopy for cleansing loose bodies and partial meniscectomy before the injection of autologous adipose tissue. Knee arthroscopy is not superior to sham surgery in OA treatment [19], but its association with the intra-articular administration of purified autologous adipose tissue has shown a benefit in pain relief and functional recovery, as reported by Caforio et al. [13], who considered that lavage and debridement could set up an ideal joint environment for subsequent cell therapies such as MSCs.

Indeed, MSCs may play a critical role in the results shown in this work. On the one hand, these cells secrete trophic, anti-inflammatory, and immunomodulatory factors (e.g., growth factors and cytokines) that promote tissue regeneration [4, 20]; on the other hand, MSCs have shown cartilage repair properties in vitro and in animal studies [6]. Therefore, the decrease in pain and improvement of functionality observed in this study could be attributable to the trophic and immunomodulation potential of MSCs.

In this analysis, the treatment is effective up until nine months. This improvement could last even longer, as reported in other studies [13, 14], but further research should be performed to confirm it.

This work has some limitations. First, it lacks a control group and the radiological assessment after the procedure. Indeed, future studies should compare the results of adipose tissue infiltration with those of traditional treatments (e.g., hyaluronic acid or PRP) and include radiological evaluations post-treatment. In addition, the analysis did not allow to differentiate the outcomes of adipose tissue infiltration according to the performance of a previous knee arthroscopy or the degree of knee OA. Although these subanalyses may have added more value to the results, they were not deemed appropriate due to the small sample size. Further studies with more patients would be of interest in order to assess the impact of the degree of OA and concomitant treatments on the pain and functionality improvement of adipose tissue infiltration. However, the aim of this study was not to assess separately the efficacy of Lipocell according to OA degrees, but to avoid the exclusion of patients based on this variable, as happens in most studies. In addition, a large proportion of patients presented with grade IV OA. Therefore, the results add evidence to the existing literature on autologous adipose tissue infiltration with Lipocell device as well as providing data about patients not usually assessed.

Joint rejuvenation is key to fight OA, and regenerative medicine can help to fill the gap between conservative treatments and joint replacement. Moreover, patients are not often willing to undergo surgery, but they are good with less invasive procedures, such as injections. In the future, if regulatory approval is obtained, allogenic and expanded MSCs could be used for the preparation of precise cell doses in vials without requiring the harvest on every single patient. However, at the moment, regulations impose some restrictions, and people could be reluctant to receive donors’ cells.

In conclusion, this study supports the safety and efficacy of the intra-articular infiltration of purified autologous adipose tissue in OA patients. If more studies confirm these findings, maybe in a few years it could become state of the art in early OA treatment.

## Data Availability

The datasets used during the current study are available from the author on reasonable request.

## Abbreviations

AD-MSCs: Adipose-derived mesenchymal stem cells
BMI: Body mass index
MSCs: Mesenchymal stem cells
OA: Osteoarthritis
PRP: Platelet-rich-plasma
VAS: Visual analogue scale
WOMAC: Western ontario and mcmaster universities osteoarthritis index
